# How do medical specialists value their own intercultural communication behaviour? A reflective practice study

**DOI:** 10.1186/s12909-016-0727-9

**Published:** 2016-08-24

**Authors:** E. Paternotte, F. Scheele, T. R. van Rossum, M. C. Seeleman, A. J. J. A. Scherpbier, A. M. van Dulmen

**Affiliations:** 1 Department of Healthcare Education, OLVG Hospital, P.O. Box 9243, 1006 AE Amsterdam, The Netherlands; 2 Medical School of Sciences, Vu University Medical Centre, P.O. Box 7057, 1007 MB Amsterdam, The Netherlands; 3 Julius Center for Health Sciences and Primary Care, University Medical Center Utrecht, P.O. Box 85500, 3508 GA Utrecht, The Netherlands; 4 Institute for Medical Education, Faculty of Health, Medicine and Life Sciences, Maastricht University, P.O. Box 616, 6200 MD Maastricht, The Netherlands; 5 NIVEL (Netherlands Institute for health services research), P.O. Box 1568, 3500 BN Utrecht, The Netherlands; 6 Department of Primary and Community Care, Radboud University Medical Centre, P.O. Box 9101, 6500 HB Nijmegen, The Netherlands; 7 Faculty of Health Sciences, University College of Southeast Norway, P.O. Box 235, 3603 Kongsberg, Drammen, Norway

**Keywords:** Intercultural communication, Generic communication, Communication skills, Communication behaviour, Reflective practice, Person-centred communication, Videotaped consultations, Interviews

## Abstract

**Background:**

Intercultural communication behaviour of doctors with patients requires specific intercultural communication skills, which do not seem structurally implemented in medical education. It is unclear what motivates doctors to apply intercultural communication skills. We investigated how purposefully medical specialists think they practise intercultural communication and how they reflect on their own communication behaviour.

**Methods:**

Using reflective practice, 17 medical specialists independently watched two fragments of videotapes of their own outpatient consultations: one with a native patient and one with a non-native patient. They were asked to reflect on their own communication and on challenges they experience in intercultural communication. The interviews were open coded and analysed using thematic network analysis.

**Results:**

The participants experienced only little differences in their communication with native and non-native patients. They mainly mentioned generic communication skills, such as listening and checking if the patient understood. Many participants experienced their communication with non-native patients positively. The participants mentioned critical incidences of intercultural communication: language barriers, cultural differences, the presence of an interpreter, the role of the family and the atmosphere.

**Conclusion:**

Despite extensive experience in intercultural communication, the participants of this study noticed hardly any differences between their own communication behaviour with native and non-native patients. This could mean that they are unaware that consultations with non-native patients might cause them to communicate differently than with native patients. The reason for this could be that medical specialists lack the skills to reflect on the process of the communication. The participants focused on their generic communication skills rather than on specific intercultural communication skills, which could either indicate their lack of awareness, or demonstrate that practicing generic communication is more important than applying specific intercultural communication. They mentioned well-known critical incidences of ICC: language barriers, cultural differences, the presence of an interpreter, the role of the family and the atmosphere. Nevertheless, they showed a remarkably enthusiastic attitude overall was noteworthy.

A strategy to make doctors more aware of their intercultural communication behaviour could be a combination of experiential learning and ICC training, for example a module with reflective practice.

**Electronic supplementary material:**

The online version of this article (doi:10.1186/s12909-016-0727-9) contains supplementary material, which is available to authorized users.

## Background

In a modern multicultural society, doctors are increasingly confronted with patients from various ethnic backgrounds. This stresses the need for effective intercultural communication (ICC) of doctors and patients. Intercultural communication has proven to be challenging for doctors, [1] which is due to differences in; language, expectations, cultural norms and values, and assumptions of the role of the family [2–4].

Intercultural communication could be described as context-specific communication [5, 6]. Previous research showed that doctors use contextual and goal driven communication during patient encounters [7]. Therefore, doctors will benefit more from context-specific communication guidelines and training than from generic guidelines and training [7]. Betancourt advised to teach doctors a practical framework with issues that arise from cultural differences instead of teaching doctors about individual cultures which could reinforce stereotyping [8].

Research on divergent expectations of doctors and patients regarding ‘good communication’ in intercultural consultations is scarce [9, 10]. Also, little evidence is available on how purposefully medical specialists use certain communication behaviour in an intercultural context [3]. It is considered advisable to examine the views of doctors regarding their intercultural communication [7, 11]. This is important since doctor-patient communication is linked to patient satisfaction and health outcomes [12–14]. Investigating the specific intercultural communication skills required from doctors, such as asking for the language proficiency or being aware of cultural differences [3], could facilitate the development of intercultural communication training in postgraduate medical education [15, 16].

In this study, we explored how doctors evaluated their own communication with native versus non-native patients. We also explored the critical incidences experienced by doctors during intercultural communication. Critical incidences are segments of the communication which are experienced as challenging. We focused on the following research questions: How do medical specialists experience intercultural communication? How purposefully do medical specialists practice intercultural communication? What do they identify as critical incidences within intercultural medical communication?

To gain insight into the participants’ thoughts regarding their intercultural communication style, we conducted interviews based on reflective practice [17, 18].

## Methods

### Reflective practice

In this reflective practice study, interviews were held after watching videotaped consultations.

Reflective practice is an introspection procedure in which videotaped situations are replayed to the participants to stimulate recall of their concurrent cognitive processes [19]. Reflective practice enables recognition of the paradigms – assumptions, frameworks and patterns of thoughts and behaviour – that shape our thinking and action [20]. Rooted in Greek philosophy, reflective practice is based on the Socratic idea of a reasoned process of weighing up the evidence to decide whether something is believed to be true or false. Socrates used a questioning technique to raise awareness among his discussion partners.

### Cultural context of the research

The study was conducted in the teaching hospital OLVG in Amsterdam, the Netherlands. OLVG hospital is known to be ‘migrant friendly’ [21], and around 70 % of the patients were not born in the Netherlands. Consequently, the doctors in this hospital are used to intercultural communication. Interviews were conducted in Dutch, and quotes were translated into English by the researchers and checked by an English editor.

### Study sample

In this study we included medical specialists of OLVG hospital in the Netherlands, a teaching hospital situated in a multicultural area of Amsterdam. We chose medical specialists because they could be described as experienced doctors and communicators. Medical specialists were recruited by email and were asked to participate if they had previously participated in an observation study in which their conversations with native and non-native patients had been videotaped, since these videotaped consultations could be used for this reflective practice interview study. In the previous observation study, various consultations of the participants were videotaped and analysed with an intercultural communication scoring list in order to find relevant skills for intercultural communication which were practiced by the participants [22]. In the present study, all doctors were native Dutch (i.e. the participants and both their parents were born in the Netherlands).

Of each of the participants, two videotaped consultations were selected, one with a native patient and one with a non-native patient. From the database with previously videotaped consultations, the interviewer selected the first videotaped consultation with a native Dutch patient and the first videotaped consultation with a non-native patient. The non-native patients were born in Morocco, Turkey, Nicaragua, Hungary, Australia, Belgium, Pakistan or Nigeria.

### Procedure

The interviewer showed previously selected prompts from the selected videotaped consultations to elicit the participant’s subjective experience in terms of beliefs, values, attitudes and considerations regarding a certain topic [18]. These prompts consisted of 5-min fragments of the two selected videotapes. The fragments that were selected by EP concerned the part where the reason for the consultation was explored, since this is pivotal for the process of the conversation. In almost all cases this topic was dealt with in the first five minutes of the videotaped consultation.

The reflective practice interviews were held between July and August 2015. The interviews took place in the participant’s own hospital. They were conducted by one interviewer (EP) and started after the participant had signed the informed consent form.

Prior to each interview, the selected 5-min fragments were shown to the participant. The interviews were semi-structured, and contained at least the following themes: differences in communication with a native versus a non-native patient, points of improvement, and the role of the medical specialist in the conversation and critical incidences defined as important aspects of ICC pointed out by doctors.

The interviews were audiotaped and transcribed verbatim. Member checking was done by sending the participants a summary of the interview and asking for confirmation. All transcripts were anonymized. All text fragments that were considered relevant to one of the research questions were coded by attaching keywords (‘codes’). To allow new insights into ICC to emerge, the coding of the interview transcripts was open and without a previously conceived coding schedule, using the program MAX-QDA. The codes were structured by means of thematic network analysis. Thematic networks are web-like illustrations that embrace the main themes of a transcript [23]. The results will be described based on the main themes (Additional file 1: Appendix A).

### Perspectives of the researchers and analysis

In this study, knowledge was constructed together with the participants. A constructivist approach was applied, meaning that multiple truths are constructed by and between people [24].

The main researcher (EP) interviewed the participants and analysed the transcripts. Since the main researcher is a clinician, the participants could talk in medical jargon during the interviews. It was explicitly explained that during the interview nothing they said could be wrong.

The transcripts were independently analysed by another researcher with a professional background in public administration (TvR). Besides, the coding of three interviews was checked by a third researcher (CS), who has a professional background in cultural competence. All three researchers are native Dutch. To check reliability, differences in the coding and selection of fragments were discussed in an iterative process until consensus was reached about the content of the codes. This consensus was achieved after 5 transcripts. After coding 9 transcripts, no new codes were derived. The second researcher (TvR) checked the coded fragments of two further transcripts. The developed coding scheme was discussed in depth with all the authors, a communication expert and two medical education experts. The involvement of researchers with different professional backgrounds provided the opportunity to discuss the various perspectives comprised in the research theme ‘intercultural communication’.

## Results

A convenience sample of the medical specialists’ specialities was selected based on their availability and willingness to participate: gynaecology (*n* = 4, 1 M/3 F), internal medicine (*n* = 5, 4 M/1 F), orthopaedic surgery (*n* = 4, 4 M) and urology (*n* = 3, 3 M). All seventeen participants agreed with the summary of the interview, except for minor changes. Additional file 2: Appendix B provides an overview of the characteristics of the patients in the videotaped consultations per interviewee.

### Generic communication and intercultural communication

Many of the participants said to experience little difference in their communication with native or non-native patients. For example, they mentioned that they needed to explain the treatment plan or asked questions to define a diagnosis. In their perception, the communication was influenced more by personal characteristics of the patients, such as assertiveness or educational level, than by the patient’s cultural background.

*I did not experience all that many differences. (C1)*

*They are all people, they are all patients, and they all want the same: they want to get rid of their problem and they want to be heard. (C13)*

When participants did mention differences between their consultations with native and non-native patients, these were mainly focussed on the explicit challenges of intercultural communication, such as the language differences.

*I try to do the same things and to treat people with respect, even if we can’t understand each other. I probably gesticulate a bit more to explain things. (C9)*

### Awareness of participants regarding intercultural communication

Participants believed that they had an open attitude and that the background of the patient did not influence their communication. Many participants seemed to be unable to indicate what effect their communication behaviour had on the patient. For example, some participants said that they adapted their explanation of the treatment plan to the level of understanding of the patient, but they had not checked if the patient understood what they had said. However, some participants mentioned certain effects; for example, they experienced that the non-verbal behaviour of patients relaxed when they started to trust the doctor.

*They see that I’m really searching for what the real problem is. And then I feel that the tension in the patient decreases. (C13)*

While assuming to have an open attitude and no assumptions, some participants did not seem to recognize that a patient’s culture might influence his or her communication, for example in expressing pain.

*If a patient screams: ‘pain everywhere!’ I just think: ‘yeah, right’, you know. Then they are not taken seriously. If the patient just tells me what the problem is, then I will listen seriously. But if the patient makes a terrible fuss, that doesn’t work for me. (C10)*

Participants found it difficult to identify the expectations of patients from different cultural backgrounds. Participants thought that it is very important to ask patients about their reasons for requesting a consultation and what specific problem they wanted to discuss. However, when they reflected on their behaviour, they realized that most of the time they did not explicitly ask this question, and they considered this to be a point of improvement for their own communication.

*It is important to check carefully what patients from a different background expect and what is important for them. (C4)*

### Patient-centred communication

Participants said that they found it important to use the same structure of their conversation when communicating with native and non-native patients. All the participants mentioned that they thought the doctor should be the leader of the conversation, which sometimes led to a directive style in their intercultural communication.

*So if we repeatedly fail to establish a good communication, but the complaint of the patient is clear, then I think I rather tend to offer a solution in a paternalistic way. (C9)*

On the other hand*,* almost all participants stated that knowing something about the patient’s background is important for establishing the right diagnosis.

*I sometimes also ask native Dutch patients where they originally came from. (C3)*

Some participants said that they tried to adapt their communication to the patient and that, as a consequence, patients were more satisfied and felt that the doctor listened to them. They considered this equally important for both native and non-native patients.

*I let the patient do most of the talking, and I only direct the communication when it is necessary. (C13)*

### Positive attitude

An overarching finding of the interviews was that almost all the participants were positive about the diversity of their patient population. Participants mentioned that they found it a challenge rather than a problem to deal with patients from different cultural backgrounds.

*This really is an extra challenge and also fun. Because many aspects of other cultures are much better than in the Netherlands… the involvement of people, the strong family ties and the readiness to help each other. We could certainly learn from this. (C8)*

## Critical incidences of intercultural communication

### Language barriers

All the participants mentioned language differences as the main cause of problems in an intercultural conversation. They experienced that the patient’s level of Dutch language proficiency determined the degree to which language was a barrier. The participants noted that although language differences can lead to misunderstandings, they may also lead to problems at a deeper level. One of the prominent problems mentioned by the participants was that nuances in the communication are lost.

*The moment you communicate more simply, it is more difficult to express empathy. For example when asking patients about their concerns. (C7)*

Participants explained that a language barrier made them adapt their communication style, for example the way they pronounced words, that they articulated more clearly, spoke more loudly or more slowly and used more non-verbal ways of communication, such as gestures.

*I notice that I change the way I speak when talking to a non-native patient. I also start to speak in broken Dutch. (C7)*

Also, some participants said that they repeated their own words more often and felt the need to check if the patient understood an explanation. This was found to be extremely important. In the eyes of the participants, patients had to be informed adequately before starting a treatment.

*When I perform an operation, the patient has to grant permission, and therefore the patient has to really understand all the information. (C10)*

Some participants said that they found it awkward or difficult to ask about a patient’s language ability, because most of the time this would become evident anyway during the conversation, or patients would start the conversation saying that their language proficiency was low.

*Because I assume that my estimation is correct, whereas that is of course an overestimation of myself. Sometimes I ended up being surprised, when I found out during the consultation or during a second visit that the patient spoke far better Dutch and understood me much better than I thought. (C11*)

### Interpreter and role of the family

The participants mentioned the use of an interpreter as an extra impediment when there was a language barrier. All participants said that a conversation with the help of an interpreter was time consuming and difficult. They found it difficult to talk to the patient through an interpreter. The participants preferred non-professional or family interpreters because they could adapt the questions more effectively to the patient’s level of understanding.

*It feels comfortable when the family does it. A family member can adapt the question to the situation of the patient, because, of course, they know the patient and understand what the patient comprehends and prefers. (C12)*

### Cultural differences

Some of the participants mentioned cultural differences as a critical aspect when communicating with non-native patients, for example when a patient refuses to look at the doctor. However, cultural differences were not considered to be as important as language barriers or levels of intelligence. Many participants did not reflect on the cultural differences and how these influenced their communication.

*I think a language barrier, a real language barrier, is much more difficult than a cultural barrier. (C8)*

In the case of cultural differences, religious differences were mentioned as another aspect that influenced the communication. For example, the Ramadan was mentioned several times as something that should be considered when communicating with Muslim patients about treatment. Participants mentioned that it was important to have some knowledge of the religions of the patients that visit a hospital.

### Atmosphere

The atmosphere of the conversation was considered to influence the communication. For example, the communication would be more business-like if the atmosphere was not relaxed. Participants experienced that it took a greater effort to put non-native patients at ease. Humour was mentioned as a possible solution for a strained conversation, which participants considered to be also applicable in conversations with non-native patients.

*On average, it takes more time and effort to establish an easy-going conversation and a certain level of trust with a non-native patient than with a Dutch patient. (C13)*

### Reflection on the communication process

Participants were enthusiastic about the method of reflective practice. The participants said they recognized their communication behaviour on the videotaped consultation as representative of their communication in daily practice. They mentioned that watching the videotapes made them aware of their behaviour and some of them formulated points of improvement for themselves. These points of improvement mainly concerned aspects of generic communication, such as not paying so much attention to the computer, not interrupting the patient or giving the patient more space to tell their story before asking questions.

*So yes, both in my attitude towards her at that moment - I think – as well as in my choice of words. I might have done that more calmly and I do think that would be more pleasant for the patient. (C15)*

Some participants mentioned a gap between training and practice. They said that their current behaviour was a result of past intercultural communication experiences and not of any training they had received during undergraduate or postgraduate medical education. Some participants mentioned that one needed to have experience as a medical doctor to be able to be aware of one’s communication behaviour.

*Certainly we have been trained in many things, but in the end it still is just a conversation in the consulting room. (C5)*

## Discussion

The aim of this reflective practice study was to explore how medical specialists experience intercultural communication (ICC), how purposefully they practice ICC and what they identify as critical incidences within ICC. We held semi-structured interviews with participants after letting them watch their own videotaped consultations, open coded the transcripts and sorted the results thematically. The videotapes were used to facilitate the participants’ reflection on their communication behaviour. Participants experienced it as valuable to watch their own videotaped consultations. The most remarkable finding was that many of the participants said they experienced hardly any differences in their communication with native or non-native patients. They mainly reflected on generic communication skills and not on intercultural communication skills. Nevertheless, the participants described the following critical incidences concerning ICC: language barriers, cultural differences, the presence of an interpreter, the role of the family and the atmosphere. At the same time, the participants expressed a remarkably enthusiastic attitude regarding communication with patients from different cultural backgrounds.

A remarkable finding is that doctors seemed to experience hardly any differences when communicating with non-native patients, except for the occasionally mentioned language barrier. The fact that doctors in our interview study found it difficult to identify differences in their own communication behaviour could indicate that they are unaware of the specific challenges of ICC and of their own communication behaviour; alternatively, it could indicate that they already are experienced intercultural communicators. The first explanation seems to be confirmed by the fact that they did not mention specific ICC skills as being important. They even found it difficult to apply specific ICC skills, such as asking for the patient’s language proficiency [3, 4], and they saw cultural differences as less important than language differences. Our finding s are in line with the results of other researchers who found that care providers may not be aware of the challenges of cultural aspects of communication [25, 26]. Besides, doctors indicated that they did not feel adequately prepared for providing effective intercultural communication [27].

The second explanation, which hypothesized that the participants already were experienced intercultural communicators, might suggest that they did not view ICC as different from communication with native patients, since they all worked in a ‘migrant friendly’ hospital. Silverman stated that for effective clinical communication, doctors need to know about communication and experience it themselves [28]. Since the medical specialists in the present study said that they had not been trained in intercultural communication, it seems more plausible that they were not completely aware of the differences in their communication with native versus non-native patients. It is therefore advisable to combine knowledge about communication and experiential learning [28].

According to the five-phase model of Van den Eertwegh et al., the first step in a learning process to change communication behaviour is confrontation with one’s own behaviour. In our study, however, confronting the participants with their own communication behaviour did not result in a deeper reflection on their communication behaviour. A possible explanation why watching the videotaped consultations did not make doctors express increased awareness of their own ICC behaviour could be that they felt unable to reflect on their own communication behaviour at a deeper level. Since becoming conscious of one’s own behaviour is the first step in any learning process, it is important to find ways to encourage experienced doctors to reflect openly on their own communication skills [29]. This reflective practice study could have provided the first steps in raising awareness regarding the communication behaviour of the participants.

The participants in our study focused mainly on the generic communication aspects and not on the intercultural communication process. This raises the question whether the generic communication skills are more important in an intercultural context than specific intercultural communication skills. Literature on intercultural communication suggest that it has a substantial overlap with patient-centred communication [1, 30–32], in which generic communication skills are geared to communicating with each patient as a person irrespective of their background. Could the results of our study indicate that using a patient-centred communication style makes it less necessary to apply the specific intercultural communication skills? [31]. Since we did not compare the two communication approaches, this could be an area for further research.

The participants in the present study described critical incidences concerning ICC that are well known in literature [3, 4, 10]. Our results add to the literature that the importance of these intercultural communication challenges is confirmed by doctors in clinical practice, which underscores the need to pay attention to these challenges in training programmes for doctors. Although this need has been established before [10, 33], there still seems to be a gap between intercultural communication experienced by doctors and ICC theory, which mainly focusses on the challenges and specific aspects of intercultural communication [31, 34, 35].

At present, communication skills training seems to be lacking in postgraduate medical education [15, 16, 36], and the participants mentioned that they did not receive any formal intercultural communication training. It is therefore advisable to develop lifelong-learning concepts for communication in health care [36]. These training modules should enable participants to master the generic communication skills as well as ICC-specific skills [7, 19].

The participants in our study mentioned the additional value of having some specific knowledge about their patients’ native cultures. However, it is considered more important to convey knowledge about the theories on how cultural differences influence intercultural communication than to offer specific knowledge about ethnic groups, since this might reinforce stereotyping [1, 37].

### Strengths, limitations and future research

The participants included in this study all worked in the same hospital, which could limit the transferability to other hospitals. Besides, this hospital is ‘migrant friendly’ [21], which means that most doctors are experienced in communicating with patients from various cultural backgrounds. The participants who work in this hospital are probably already more adept in dealing with the influences of culture on the communication than doctors who work in hospitals with a smaller variety of cultures. On the other hand, since our findings show that even extensive experiences with ICC alone do not necessarily make medical specialists aware of the differences in their communication performance, this is likely to be true as well for the broader medical specialist population. Possibly, achieving awareness of communication behaviour requires a combination of experience and ICC training, preferably in a module with reflective practice. The effect of a combination of experience with non-native patients and intercultural communication training could be researched in more detail.

Although the professional background of the researchers all differed, a limitation could be the native status of the whole research team. Another possible limitation could be the method of semi-structured interviews with open questions. The participants were not directed into the reflection of their ICC behaviour, which could have caused that participants felt obliged to focus on the generic communication instead of the intercultural communication. On the other hand, this shows the focus of doctors regarding their communication even in an intercultural conversation.

### Practical implications for medical education

The results of this study indicate that intercultural communication experiences alone do not make a medical specialist aware of the differences between communication with native and non-native patients. Possibly, achieving awareness of communication behaviour requires a combination of experience and ICC training, rooted in patient-centred communication, preferably in a module with reflective practice.

## Conclusion

Despite extensive experience in intercultural communication, the participants of this study noticed hardly any differences between their own communication behaviour with native and non-native patients. This could mean that they are unaware that consultations with non-native patients might cause them to communicate differently than with native patients. The reason for this could be that medical specialists lack the skills to reflect on the process of the communication. The participants focused on their generic communication skills rather than on specific intercultural communication skills, which could either indicate their lack of awareness, or demonstrate that practicing generic communication is more important than applying specific intercultural communication. They mentioned well-known critical incidences of ICC: language barriers, cultural differences, the presence of an interpreter, the role of the family and the atmosphere. Nevertheless, they showed a remarkably enthusiastic attitude overall was noteworthy.

## Additional files

Additional file 1: Appendix A. List of codes derived from the transcripts. (DOCX 66 kb) Additional file 2: Appendix B. Overview of patient characteristics per interviewee. (DOCX 80 kb)

## Abbreviations

ICC: intercultural communication
