# Supplementary material for: How do medical specialists value their own intercultural communication behaviour? A reflective practice study
Source: BMC Med Educ. 2016 Aug 24;16(1):222. doi: 10.1186/s12909-016-0727-9 (PMC4997670; doi:10.1186/s12909-016-0727-9)
Supplement: Additional file 1: — Appendix A. List of codes derived from the transcripts. (DOCX 66 kb) [file 12909_2016_727_MOESM1_ESM.docx]

Additional file 1: Appendix A. List of codes derived from the transcripts.

| Reflection |
| --- |
| Unconscious behaviour |
| Conscious behaviour |
| Role of the doctor |
| Doctors’ assumptions about patients |
| Verbal communication |
| Non-verbal communication |
| Structure of the conversation |
| Leader of the conversation |
| Professional attitude of the doctor |
| Communication in medical education |
| Explaining |
| Point of improvement |
| Time |
| Atmosphere |
| Personal communication |
| Social component of communication |
| Background of the patient |
| Different communication with native and non-native patient |
| Language proficiency |
| Interpreter |
| Cultural differences |
| Cultural diversity as part of the job |
| Patient-centred |
| Role of the family |
| Doctor-patient relation |
| Goal of the conversation |
| Listening |
| Taking the patient seriously |
| Consequences of language barrier |
| Education level of patient |
| Generation level of immigration of the patient |
| The feeling of being understood |
| Trust |
| Computer |
| The speed of talking |
| Articulation |
| Expectations of the patient |
| Greeting |
| Empathy |
| Summarising |
| Humour |
| Open attitude |
| Loud voice |
| Preferences of doctors |
| Respect |
| Misunderstanding |
| Medical jargon |
| Taking decisions |
| Patient satisfaction |
| Patient-autonomy |
| Reassuring the patient |
