# Supplementary material for: How do medical specialists value their own intercultural communication behaviour? A reflective practice study
Source: BMC Med Educ. 2016 Aug 24;16(1):222. doi: 10.1186/s12909-016-0727-9 (PMC4997670; doi:10.1186/s12909-016-0727-9)
Supplement: Additional file 2: — Appendix B. Overview of patient characteristics per interviewee. (DOCX 80 kb) [file 12909_2016_727_MOESM2_ESM.docx]

Additional file 2: Appendix B. Overview of patient characteristics per interviewee.

| Code of interview | Duration of the interview (minutes) | Patient ethnicity* | Dutch language proficiency of patients** | Informal interpreter (yes/no), (companion) |
| --- | --- | --- | --- | --- |
| C7 | 31 min | Dutch | Good | No (with partner) |
|  |  | Afghan | Moderate | Yes (partner) |
| C8 | 33 min | Nicaraguan | Good | No, alone |
|  |  | Dutch | Good | No (with partner) |
| C15 | 25 min | Dutch | Good | No (with daughter) |
|  |  | Turkish | Good | No, alone |
| C1 | 31 min | Turkish | Moderate | No, alone |
|  |  | Dutch | Good | No, alone |
| C5 | 43 min | Hungarian | Good | No, alone |
|  |  | Dutch | Good | No, alone |
| C16 | 25 min | Dutch | Good | No, alone |
|  |  | Turkish | Good | No, alone |
| C3 | 15 min | Dutch | Good | No (with partner) |
|  |  | Turkish | Good | No (with partner) |
| C9 | 25 min | Dutch | Good | No, Alone |
|  |  | Australian | Bad, patient speaks English | No, conversation in English (with partner) |
| C4 | 25 min | Dutch | Good | No, alone |
|  |  | Nigerian | Bad | No, conversation in English (with partner and child) |
| C14 | 26 min | Dutch | Good | No, with child |
|  |  | Turkish | Good | No, alone |
| C13 | 31 min | Moroccan | Good | No, alone |
|  |  | Dutch | Good | No (with partner) |
| C17 | 29 min | Dutch | Good | No, alone |
|  |  | Moroccan | Good | No, alone |
| C10 | 31min | Moroccan | Moderate | No, alone |
|  |  | Dutch | Good | No alone |
| C2 | 29 min | Dutch | Good | No, alone |
|  |  | Turkish | Bad | Yes, daughter |
| C6 | 20 min | Dutch | Good | No, alone |
|  |  | Moroccan | Moderate | No, alone |
| C11 | 27 min | Pakistani | Bad | Yes, daughter |
|  |  | Dutch | Good | No, alone |
| C12 | 37 min | Dutch | Good | No, alone |
|  |  | Belgian | Moderate | Yes, partner (with child) |

*The interval of the age of the patients was 22-79 years. The mean age was 48 years.

**Based on the authors opinion and trustworthiness checked with the interviewed doctor, 100% similar.
